# Anti-Xa activity after subcutaneous administration of dalteparin in ICU patients with and without subcutaneous oedema: a pilot study

**DOI:** 10.1186/cc4952

**Published:** 2006-06-21

**Authors:** Mirjam K Rommers, Netty Van Der Lely, Toine CG Egberts, Patricia MLA van den Bemt

**Affiliations:** 1 Hospital Pharmacy Midden-Brabant; TweeSteden Hospital and St Elisabeth Hospital, Tilburg, the Netherlands; 2 Department of Clinical Pharmacy and Toxicology, Leiden University Medical Centre, Leiden, the Netherlands; 3 Department of Intensive Care, St Elisabeth Hospital, Tilburg, the Netherlands; 4 Department of Pharmaco-epidemiology and Pharmacotherapy Utrecht, Institute for Pharmaceutical Sciences, Utrecht University, the Netherlands

## Abstract

**Introduction:**

Intensive care unit (ICU) patients often suffer from subcutaneous oedema, due to administration of large fluid volumes and the underlying pathophysiological condition. It is unknown whether the presence of subcutaneous oedema impairs the absorption of dalteparin, a low molecular weight heparin, when it is given by subcutaneous administration for venous thromboembolism prophylaxis. The objective of this study is to compare the anti-Xa activity of dalteparin after subcutaneous administration in ICU patients with and without subcutaneous oedema.

**Methods:**

This non-randomized open parallel group follow-up pilot study was conducted in two mixed medical-surgical intensive care units at two teaching hospitals. Seven ICU patients with subcutaneous oedema (index group) and seven ICU patients without subcutaneous oedema (reference group) were studied. Anti-Xa activity was determined at 0, 3, 4, 6, 8, 12 and 24 hours after subcutaneous administration of 2,500 IU dalteparin. Plasma concentrations of factor anti-Xa activity were measured using a chromogenic factor Xa inhibition assay.

**Results:**

The characteristics of the index group were: age, 58 years; male/female ratio, 5/2; body mass index at admission, 23.4 kg/m^2 ^(at study day, 30.6 kg/m^2^). The characteristics of the reference group were: age, 49 years; male/female ratio, 6/1; body mass index at admission, 24.8 kg/m^2 ^(at study day, 25.0 kg/m^2^). In the index group, creatinine clearance was lower compared to the reference group (71 versus 131 ml/minute, *p *= 0.003). Sequential organ failure assessment score did not differ between index and reference groups (4 versus 5). Mean arterial pressure was comparable between index and reference groups (91 versus 95 mmHg) and within the normal range. The mean C_max _value was not different between ICU patients with and without subcutaneous oedema (0.15 ± 0.02 versus 0.14 ± 0.02 IU/ml, *p *= 0.34). In the index group, the mean AUC_(0–24 h) _value was slightly higher compared with the reference group (1.50 ± 0.31 versus 1.15 ± 0.25 h·IU/ml, *p *= 0.31). This difference was not significant.

**Conclusion:**

In this pilot study, there was no clinically relevant difference in anti-Xa activity after subcutaneous administration of 2,500 IU dalteparin for venous thromboembolism prophylaxis between ICU patients with and without subcutaneous oedema. Critically ill patients seem to have lower anti-Xa activity levels than healthy volunteers.

## Introduction

Venous thromboembolism (VTE) is a frequent (10% to 80%) complication in critically ill patients admitted to intensive care units (ICUs) [1,2]. Critically ill patients have a higher risk of VTE due to several risk factors such as increased age, recent surgery, venous stasis as a result of prolonged immobilization, acute infectious disease, hypercoagulability resulting from acute phase responses, and vascular injury caused by central venous catheters or other invasive interventions [1-3]. Most ICU patients therefore receive thromboprophylaxis with mechanical methods, unfractionated heparin or subcutaneous low molecular weight heparins (LMWHs) [2,4,5]. Several randomized clinical trials and meta-analyses have demonstrated that subcutaneous LMWHs are efficient and safe in the prevention of VTE in surgical and medical patients [6-10]. Trials in ICU patients have, however, rarely been conducted.

Patients in the ICU with shock symptoms often require large volumes of fluid to maintain perfusion and thereby tissue oxygenation and to prevent multi-organ dysfunction syndrome. Due to the administration of large volumes of fluid as well as the underlying pathophysiological condition, ICU patients often suffer from substantial subcutaneous oedema.

A number of factors might interfere with the effectiveness of subcutaneous administrated LMWHs in critically ill patients, such as low cardiac output, decreased peripheral blood flow, use of vasopressors or subcutaneous oedema [11-14]. Subcutaneous oedema may impair the absorption of medication given by subcutaneous injection [15]. We postulate that the absorption of subcutaneous dalteparin, a LMWH used for thromboprophylaxis in our ICU, is impaired in patients with subcutaneous oedema. This possible impairment may be due to either a delayed absorption or to a reduced absorption.

Because it is difficult to measure LMWH concentrations directly, pharmacokinetic studies generally use surrogate biological effect markers such as anti-Xa activity [16-22], which has been shown to be correlated with the administrated dose as well as, although more controversial, the clinical effect [23-25].

To investigate whether indeed the absorption of dalteparin is impaired in ICU patients with subcutaneous oedema, we compared anti-Xa activity after subcutaneous injection of dalteparin in ICU patients with subcutaneous oedema with anti-Xa activity in ICU patients without subcutaneous oedema.

## Materials and methods

This non-randomized open parallel group follow-up pilot study was performed in the ICUs of the St Elisabeth Hospital and the TweeSteden hospital in Tilburg, the Netherlands, from January 2003 until July 2005. Both ICUs served medical as well as surgical patients. The medical ethics committee of the St Elisabeth Hospital approved the study protocol for both hospitals.

Inclusion criteria were ICU patients with age >18 years and subcutaneous administration of dalteparin 2,500 IU once daily for VTE prophylaxis. Exclusion criteria were concurrent use of vitamin K antagonists, use of therapeutic doses of unfractionated heparin or LMWHs, severe liver failure (bilirubin >40 μmol/l), renal insufficiency (creatinine clearance <30 ml/minute), signs of disseminated intravascular coagulation (platelets <100 × 10^9^/l, prolonged prothrombin time, and activated partial thromboplastin time), use of vasopressors and/or inotropics. All patients or their legal representatives gave informed consent before actual inclusion. After inclusion, the measurements took place on a day the patient had used dalteparin in the ICU unit for at least three days.

Two groups of patients were studied: ICU patients with subcutaneous oedema (index group) and ICU patients without subcutaneous oedema (reference group). Subcutaneous oedema was defined as a weight gain of at least 10% compared to the weight of the patient at admission and an appearance of substantial generalized subcutaneous oedema. This definition was chosen because we needed a measurement that was easy to use in common practice and, to our knowledge, there is no validated definition or measurement method for oedema in ICU patients. Patients were weighed by means of a 'weighing-bed'.

All patients received a subcutaneous injection of dalteparin (Fragmin^®^, Pharmacia BV, Woerden, the Netherlands) that was administrated by the nursing staff of the ICU using prefilled single-dose syringes (0.2 ml = 2,500 IU). The same technique and injection site, the abdomen or upper thigh, was used in every patient.

Primary endpoint was the difference in anti-Xa activity between index and reference group. On the study day, blood samples were obtained immediately before and 3, 4, 6, 8, 12 and 24 hours after administration of dalteparin. Blood was drawn by venipuncture. Each time 5 ml blood was collected in sodium citrate (0.109 M, 3.2%) tubes and sent to the laboratory as soon as possible. There, plasma was separated from cells by centrifugation (3,000 rpm for 10 minutes) and plasma was stored at -20°C for a maximum period of one month. Plasma concentrations of factor anti-Xa activity were measured with an ACL3000 instrument (Instrumentation Laboratory BV, Breda, The Netherlands) using a chromogenic factor Xa inhibition assay (Spectrolyse^®^Heparin Xa, Trinity Biotech, Ireland). The anti-Xa assay standard calibration curve of dalteparin ranged from 0.0 IU/ml to 0.8 IU/ml. The within assay and among assay precision (coefficient of variation) was, for 0.1 IU/ml heparin, 8.0% and 8.9%, respectively, and, for 0.5 IU/ml heparin, 3.7% and 6.6%, respectively.

A concentration-time curve of anti-Xa activity was determined for all patients. For each patient, the C_max_, the maximum observed activity, was estimated from the anti-Xa concentration curve and the AUC_(0–24 h)_, the area under the concentration curve at 0 to 24 hours, was calculated by the trapezoidal rule. Of these measurements, we calculated the mean plasma concentrations of anti-Xa activity and mean C_max _and AUC_0–24 h _for both groups.

In addition, the following data were collected from the medical history of the patient: age, sex, weight (at admission and at study day), length, sequential organ failure assessment (SOFA) score at study day, and mean arterial pressure (MAP) at study day and diagnosis. On the study day, urine output over a 24 hour period was collected for determination of the creatinine clearance.

We calculated that seven evaluable patients were needed in each group to prove a difference of 50% in anti-Xa activity. We considered a difference of 50% in anti-Xa activity, in either AUC_0–24 h _or C_max_, to be clinically relevant. This was based on the assumption of a C_max _in healthy volunteers of 0.22 (standard deviation (SD) 0.07) IU/ml and an AUC_0–24 h _of 1.26 (SD 0.40) h·IU/ml [17] and a desired power of 80% and α of 5%.

Comparison of continuous variables was done by Students *t *test. The anti-Xa activity (C_max _and AUC_(0–24 h)_) was compared by the non-parametric Mann-Whitney test (mean ± SD). A *p *value of 0.05 is taken as cut-off for statistical significance.

## Results

During the study period, all patients at the ICUs were screened to fulfill inclusion criteria. Most patients were excluded because they were on vasopressor or inotropic medication. Finally, seven patients in the index group and seven patients in the reference group were included. Demographic and clinical characteristics of the patients are listed in Table 1. The distribution of age, sex, length, weight and body mass index (at admission) were not different between the two groups. All patients received respiratory support. Weight and body mass index at study day was higher, as expected, in the index group, because of the at least 10% weight gain in patients with subcutaneous oedema that was required for inclusion. In ICU patients with subcutaneous oedema, creatinine clearance was lower compared with ICU patients without subcutaneous oedema (71 ml/minute versus 131 ml/minute, *p *= 0.003). The SOFA score did not differ between the index and the reference group. MAP, used as an estimate of adequacy of tissue perfusion, was within the normal range in both groups (80 to 100 mmHg). The diagnoses varied between the two groups. Patients in the index group had a diagnosis of sepsis more often (four times) than patients in the reference group (zero times). As mentioned, none of the patients used vasopressors or inotropics.

**Figure 1:**
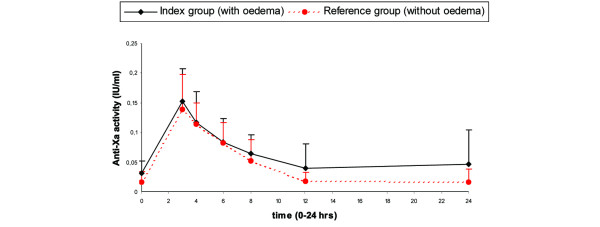
Mean anti-Xa activity concentration (+ standard deviation) of the index group and the reference group (IU/ml).

The results of anti-Xa activity measured for the two groups are given in Table 2 and Figure 1. Peak anti-Xa activity (C_max_) is the anti-Xa activity three hours after administration of dalteparin (Figure 1). Mean C_max _and AUC_(0–24 h) _values are listed in Table 3. The mean C_max _value was not different between ICU patients with and without subcutaneous oedema (0.15 IU/ml and 0.14 IU/ml, respectively; *p *= 0.34). In the index group the mean AUC_(0–24 h) _value was slightly higher compared with the reference group (1.50 h·IU/ml versus 1.15 h·IU/ml, *p *= 0. 31). This difference is not significant.

## Discussion

For non-intravenous, parenteral routes of drug administration, such as subcutaneous injection, decreased peripheral (cutaneous) blood flow, changes in local pH, oedema and scar tissue may alter the extent of absorption [14]. In one study the absorption of subcutaneous administrated insulin was significantly lower and delayed in patients with generalized subcutaneous oedema [15]. Therefore, we considered it possible that the absorption of dalteparin, administrated by subcutaneous injection for prophylaxis of VTE, might be influenced by subcutaneous oedema, very often seen in ICU patients with shock symptoms. In this study, we cannot confirm this hypothesis. Mean plasma concentrations of anti-Xa activity are comparable in ICU patients with and without subcutaneous oedema.

The pharmacokinetics of dalteparin is linear and dose-independent for anti-Xa activity. Absorption is rate-limiting after subcutaneous administration and peak plasma concentrations are attained after 2.8 to 4 hours. Bioavailability after subcutaneous injection is close to 90%. The apparent volume of distribution is close to the plasma volume. Elimination appears to occur mainly via the renal route, with a plasma elimination half-life of 2.4 to 4 hours [17,18]. The mean C_max _values of 0.15 IU/ml and 0.14 IU/ml found in the index and the reference groups, respectively, are lower than the assumed C_max _value in healthy volunteers of 0.22 IU/ml. The mean AUC_(0–24 h) _value of 1.15 h·IU/ml in ICU patients without subcutaneous oedema is also lower than the demonstrated AUC_(0–24 h) _value in healthy volunteers (1.26 h·IU/ml) [17]. This can be an indication of lower anti-Xa activity levels in critically ill patients. Others find this as well [11,12,26,27]. Dörffler-Melly [11] and colleagues demonstrated lower anti-Xa activity in ICU patients on vasopressors. The use of vasopressors, they explain, leads to adrenergic vasoconstriction of the peripheral blood vessels and thus impaired perfusion of the subcutaneous tissue. This was the reason to exclude patients on vasopressors and/or inotropics from our study. Priglinger and colleagues [12] showed lower anti-Xa levels in critically ill patients compared with medical patients in the normal ward. They did not find a correlation between the dose of norepinephrine and the anti-Xa activity. Priglinger and colleagues [12] and Freedman [13] mention altered cardiac output, pre-existing conditions and also use of vasopressors and decreased peripheral blood flow as possible reasons for lower anti-Xa activity in ICU patients. Mayr and colleagues [26] demonstrated very low anti-Xa levels in intensive care patients, especially in patients with high multi-organ dysfunction syndrome score and high body weight. And a recent study by Rutherford and colleagues [27] showed subtherapeutic trough levels of anti-Xa activity in critically ill trauma and surgical patients after once daily 40 mg enoxaparin by subcutaneous injection for deep venous thrombosis prophylaxis.

Critical illness may alter absorption, volume of distribution and clearance of drugs used in ICU patients and these alterations may have additive or opposing effects on drug exposure, elimination and half-life [14]. In contrast to unfractionated heparin, only a minor portion of LMWH binds to acute phase proteins and endothelial cells. Whether the bioavailability of LMWHs decreases during an acute phase response, as it can be found in critically ill patients, is unknown [12,26]. These and other combinations of complex pathophysiological characteristics of the ICU patient may account for the lower anti-Xa concentration in our ICU patients.

In our study, the two groups are well matched in terms of age, sex, length, weight and body mass index at admission. As there was no difference in MAP between the two groups, tissue hypoperfusion was not a confounding variable. The SOFA score (0 to 24) is useful to assess organ dysfunction or failure and to evaluate morbidity. It can be used to characterize groups of patients for comparison in trials [28,29]. The SOFA scores were equal in the index and reference groups, indicating comparability in morbidity between both groups. The two groups do not match in terms of creatinine clearance. The creatinine clearance in the index group is lower compared to the reference group (71 ml/minute versus 131 ml/minute). LMWHs can accumulate in patients with renal failure. It is known from the literature that renal insufficiency does not have any influence on C_max _and apparent volume of distribution of LMWHs, but significantly prolongs clearance and increased half-life [16,19,30,31]. It looks like the elimination half-life was shorter in the reference group compared to the index group (Figure 1). This may have contributed to the slightly higher AUC_(0–24 h) _in the index group. However, no patients had a creatinine clearance of less than 30 ml/minute. The difference in AUC_(0–24 h) _is more likely due to the large inter-patient variability in anti-Xa concentrations after subcutaneous administration of 2,500 IU dalteparin (Table 2). The measurements of anti-Xa activity are in the lower part of the calibration curve; some of the data are below the detection limit (0.10 IU/ml). The reliability of the assay might be less in this part of the curve and may have contributed to the large inter-patient variability in the data and so to the higher AUC_(0–24 h) _in the index group. The AUC_(0–12 h) _and AUC_(0–8 h)_, in which the lower concentrations play a less important role, were more comparable between the index and the reference group (0.90 and 0.71 IU/ml for the index group and 0.75 and 0.63 IU/ml for the reference group, respectively). High variability among patients in anti-Xa activity, however, has been reported by others [31]. The complex pathophysiological characteristics of the ICU patient may have played a roll in this as well.

When interpreting the results of this study, some other points must be taken into consideration. This is a pilot study with only a small number of patients. A relatively long inclusion period was needed to meet enrollment of the required number of patients not on vasopressors and/or inotropics. With our sample size calculation we considered a difference of 50% in anti-Xa activity to be clinically relevant. To prove that difference, we only needed a small number of patients, seven in each group. With a larger number of patients we might have been able to prove a less than 50% difference in anti-Xa activity between the two groups.

Although commonly used to monitor anticoagulant effectiveness of LMWHs, lower anti-Xa levels do not necessarily mean that critically ill patients are insufficiently protected against VTE using the subcutaneous route of administration [23-25]. To adequately answer that question would take a much larger efficacy study with a different endpoint, such as the presence of VTE instead of anti-Xa levels. Besides this, there are other mechanisms by which LMWHs influence haemostasis. These other mechanisms include inhibition of thrombin activity, inhibition of platelet function and, possibly, enhancement of fibrinolytic activity. Thus, anti-Xa activity may be a marker of LMWHs, which may or may not be causally related to the clinical outcomes [13,23,25]. It will be interesting to investigate, as Freedman also mentioned, the intravenous route of administration of LMWHs for thromboprophylaxis in critically ill patients and determine anti-Xa activity and efficacy [13].

Besides these limitations, this study is, to our knowledge, the first to investigate the influence of subcutaneous oedema on the absorption of LMWHs in the critically ill patient. Subcutaneous oedema is a common problem in the ICU patient. Notwithstanding the difficult patient population, we have managed to include comparable ICU patients in both groups. ICU patients have a complex pathophysiology that can influence the pharmacology of different drugs. It is important to investigate parts of this potential problem so we can optimize the use of drugs in critically ill patients.

## Conclusion

In this pilot study there was no clinically relevant difference in anti-Xa activity after subcutaneous administration of 2,500 IU dalteparin for VTE prophylaxis between ICU patients with and without subcutaneous oedema. Critically ill patients, however, seem to have lower anti-Xa activity levels than healthy volunteers. Whether these lower anti-Xa activities also translate into higher prevalence of thromboembolic events is still unclear. Further studies are necessary to identify the proper dose and route of application of LMWHs for VTE prophylaxis in the critically ill patient.

## Key messages

• 	ICU patients often suffer from subcutaneous oedema and it is unknown whether this oedema impairs the absorption of LMWHs given by subcutaneous injection.

• 	We found no difference in anti-Xa activity after subcutaneous administration of 2,500 IU dalteparin for VTE prophylaxis between ICU patients with and without oedema.

• 	Critically ill patients seem to have lower anti-Xa activity levels than healthy volunteers.

## Abbreviations

AUC = area under the concentration curve; C_max _= maximal observed activity; ICU = intensive care unit; LMWH = low molecular weight heparin; MAP = mean arterial pressure; SD = standard deviation; SOFA = sequential organ failure assessment; VTE = venous thromboembolism.

## Competing interests

The authors declare that they have no competing interests.

## Authors' contributions

MR participated in design and coordination of the study and drafted the manuscript. NL participated in design and coordination of the study and helped to draft the manuscript. TE participated in design of the study and helped to draft the manuscript. PB participated in design of the study and helped to draft the manuscript. All authors read and approved the final manuscript.

## Figures and Tables

**Table 1:** Demographic and clinical characteristics of the patients

	Index group with oedema (*n *= 7)	Reference group without oedema (*n *= 7)	*P *value
Age (years)	58 (32–85)	49 (21–71)	0.39
Men	5 (71%)	6 (86%)	
Length (cm)	171 (165–185)	176 (165–185)	0.26
Weight at admission (kg)	68 (47–80)	77 (65–90)	0.20
Weight at study day (kg)	89 (65–107)	77 (65–85)	0.09
Weight gain (kg)	21 (31%)	1 (1%)	0.001
BMI at admission (kg/m^2^)	23.4 (16.3–27.6)	24.8 (21.0–30.4)	0.47
BMI at study day (kg/m^2^)	30.6 (22.5–37.9)	25.0 (21.5–30.4)	0.04
SOFA score	4 (3–5)	5 (3–6)	0.41
Creatinine clearance (ml/minute)	71 (36–109)	131 (85–168)	0.003
Mean arterial pressure (mmHg)	91 (80–115)	95 (79–122)	0.64
Diagnosis (number)			
Sepsis	4	0	
Neurotrauma	1	2	
Intoxication	1	0	
Pneumonia	0	1	
Multitrauma	0	2	
Exacerbation COPD	0	1	
Perforated appendicitis	1	0	
Cerebral bleeding	0	1	

Data are means with ranges in parentheses unless otherwise specified. BMI, body mass index; COPD, chronic obstructive pulmonary disease; SOFA, sequential organ failure assessment.

**Table 2:** Anti-Xa activity before (0 h) and 3, 4, 6, 8, 12 and 24 h after subcutaneous administration of 2,500 IU dalteparin

	Anti-Xa activity (IU/ml)							
	
	0 h	3 h	4 h	6 h	8 h	12 h	24 h	AUC_(0–24 h)_
Index: with oedema								
1	0.01	0.18	0.14	0.03	0.06	0.02	0.01	1.05
2	0.00	0.20	0.17	0.08	0.03	0.00	0.00	0.91
3	0.02	0.15	0.11	0.10	0.04	0.03	0.06	1.42
4	0.06	0.16	0.14	0.12	0.09	0.02	0.04	1.53
5	0.03	0.04	0.02	0.03	0.03	0.02	0.02	0.59
6	0.00	0.14	0.12	0.12	0.10	0.12	0.17	2.98
7	0.10	0.20	-^a^	0.11	0.10	0.07	0.03	2.07
Mean (SD)	0.03 (0.02)	0.15 (0.05)	0.12 (0.05)	0.08 (0.04)	0.06 (0.03)	0.04 (0.04)	0.05 (0.06)	1.50 (0.81)
Reference: without oedema								
1	0.00	0.09	0.08	0.05	0.01	0.00	0.00	0.43
2	0.01	0.14	0.13	0.09	0.07	0.03	0.02	1.24
3	0.00	0.25	0.16	0.08	0.03	0.00	0.00	0.99
4	0.01	0.10	0.09	0.07	0.05	0.03	0.00	0.88
5	0.04	0.08	0.09	0.09	0.05	0.03	0.02	1.05
6	0.02	0.13	0.09	0.05	0.04	0.02	0.02	0.93
7	0.04	0.18	0.16	0.15	0.12	-^a^	0.06	2.52
Mean (SD)	0.02 (0.02)	0.14 (0.06)	0.11 (0.04)	0.08 (0.03)	0.05 (0.03)	0.02 (0.01)	0.02 (0.02)	1.15 (0.65)

^a^Missing value. AUC, area under the concentration curve; SD, standard deviation.

**Table 3:** Mean C_max _and AUC_(0–24 h) _anti-Xa activity

	Index group with oedema (*n *= 7)	Reference group without oedema (*n *= 7)	*P *value
C_max _(IU/ml)	0.15 ± 0.02	0.14 ± 0.02	0.34
AUC_(0–24 h) _(h·IU/ml)	1.50 ± 0.31	1.15 ± 0.25	0.31

Data are means ± standard error of the mean. AUC, area under the concentration curve; C_max_, maximal observed activity.
